# Strategies and Challenges in Recruiting Pregnant Women with Elevated Body Mass Index for a Behavioral Lifestyle Intervention

**DOI:** 10.1089/whr.2020.0089

**Published:** 2020-12-07

**Authors:** Jihong Liu, Sara Wilcox, Ellen Wingard, Judith Burgis, Lara Schneider, Alicia Dahl

**Affiliations:** ^1^ Department of Epidemiology and Biostatistics and Arnold School of Public Health, University of South Carolina, Columbia, South Carolina, USA.; ^2^ Department of Exercise Science, Arnold School of Public Health, University of South Carolina, Columbia, South Carolina, USA.; ^3^ Prevention Research Center, Arnold School of Public Health, University of South Carolina, Columbia, South Carolina, USA.; ^4^ Department of Obstetrics and Gynecology, Prisma Health-USC Medical Group, Columbia, South Carolina, USA.; ^5^ Department of Public Health Sciences, University of North Carolina at Charlotte, Charlotte, North Carolina, USA.

**Keywords:** pregnancy, recruitment, maternal obesity, technology, randomized controlled trial

## Abstract

***Purpose:*** Pregnant women with elevated body mass index (BMI) are difficult to recruit into lifestyle studies. This article (1) summarized strategies to recruit pregnant women into a randomized trial, and (2) reported recruitment statistics and their correlates.

***Materials and Methods:*** African American and white women with BMI ≥25 and gestational age <16 weeks were recruited primarily through obstetric clinics into the Health in Pregnancy and Postpartum study. Women completed a brief screening form, and if initially eligible, a phone screening. We compared characteristics of those randomized versus not randomized.

***Results:*** Initially eligible pregnant women (*N* = 1578) were identified through direct recruitment by research staff, indirect recruitment by clinic staff at obstetric clinics, and self-referrals through advertisements. Of these women, 54.0% (850) were reached for further screening, and 43.5% (685) were fully eligible. Among eligible women, 58.8% (403) were scheduled for a baseline visit, and 33.3% (228) were randomized. The overall recruitment yield was 14.4%. Recruited participants were diverse (44% African Americans) and averaged 12.6 weeks gestation at baseline. Randomized (vs. nonrandomized) women were more likely to own a cell phone, have access to a computer with internet at home or work, and have downloaded a podcast.

***Conclusions:*** Although this study did not reach the recruitment goal, a relatively large and diverse sample of pregnant women were recruited early in pregnancy. Recruiting women with elevated BMI for a behavioral lifestyle intervention is challenging, particularly among women with characteristics, including less phone and internet access and limited experience in using podcasts.

This study is registered at Clinicaltrials.gov: NCT02260518.

## Introduction

Emerging evidence has shown that pregnancy is an important period for chronic disease prevention for both the mothers and their offspring.^1–5^ Pregnancy is often regarded as a “teachable moment” for health behavior change as women are more open to health advice due to their increased motivation to maximize their own health and that of their unborn child.^6,7^ The number of pregnancy studies has increased dramatically in the past two decades. For example, a PubMed search conducted in November 2019 for terms “pregnancy” and “randomized controlled trial” and restricting to human studies revealed 1446 articles in 2018, compared with 531 articles in 2000.

Over two-thirds (68.9%) of U.S. women are either overweight or obese, and the percentage who are overweight or obese among non-Hispanic African American women is even higher (80%).^8,9^ Close to one-half (47.2%) of all pregnant U.S. women exceed the Institute of Medicine (IOM)-recommended weight gain^10,11^ and the trend of gaining above IOM recommendations appears to be rising.^12,13^ Overweight and obese women are at even greater risk for excessive gestational weight gain (GWG) with nearly two-thirds (64%) exceeding recommendations.^11^ GWG is a modifiable risk factor for several adverse maternal and neonatal outcomes.^14^ Thus, maternal obesity and excessive weight gain have received increasing attention among researchers as illustrated by an increase in the collective number of behavioral lifestyle intervention studies being published over time.^15–18^

Despite the relatively large number of behavioral intervention studies in overweight or obese pregnant women, most are underpowered due to small sample sizes^17^ and have failed to meet recruitment goals.^19,20^ For example, LIFE-Moms (Lifestyle Interventions for Expectant Moms) is an NIH-funded consortium of seven independent but collaborative clinical trials designed to evaluate the efficacy of varied lifestyle intervention programs to ameliorate excessive GWG among overweight and obese pregnant women. Three out of seven sites in LIFE-Moms were only able to recruit 31 to 54 eligible women into their randomized controlled trials, which was far below their enrollment target ranging from 200 to 400 participants.^19,21^

The body of literature suggests that pregnant women, and particularly overweight and obese pregnant women, are difficult to recruit into intervention studies and may require unique approaches. Studies that report recruitment statistics and strategies on how to successfully recruit pregnant women into intervention studies could help researchers better plan their recruitment and study timelines and enhance their recruitment efficiency. Thus, using the data from a recently completed randomized controlled trial among overweight and obese pregnant women (described later), the purpose of this article was to report recruitment strategies and yield, compare the characteristics of those who were randomized with those who were not, and share learnings in recruiting high-risk pregnant women.

## Materials and Methods

The Health in Pregnancy and Postpartum (HIPP) study is a randomized controlled trial assessing the efficacy of a behavioral intervention during pregnancy and postpartum on adequate GWG, postpartum weight loss, improved health behaviors, quality of life, and favorable offspring body composition. A sample of 400 women was planned to detect a 2.0 kg difference in total GWG between intervention and standard care participants, corresponding to an effect size of 0.28, assuming a two-sided type I error rate of 0.05 and 80% power. Equal numbers of white and African American (*n* = 200, each) were planned to examine the racial differences in intervention's effects on GWG. Our main project site (Richland county) had a high proportion of African Americans (48.7%).^22^ Participants were recruited from the Midlands area of South Carolina from January 1, 2015 through December 31, 2018. Study visits were conducted at ≤16 weeks gestation, 32 weeks gestation, 6 months postpartum, and 12 months postpartum.

Participants in the behavioral intervention group received two in-depth counseling sessions (one each in early pregnancy and early postpartum), weekly or biweekly telephone counseling, 10 weekly behavioral podcasts with accompanying educational handouts on nutrition and physical activity during pregnancy, and access to a private Facebook group to interact with other study participants. Podcasts (10 in pregnancy, 16 in postpartum) were used to reinforce behavioral principles covered in the counseling calls. The intervention was based on Social Cognitive Theory.^23^ Participants in the standard care group attended regularly scheduled clinic visits with their prenatal care providers, received monthly study mailings, and received podcasts (same schedule as intervention participants) focusing on tips for healthy pregnancy, fetal or infant development, and parenting. More details of HIPP study were described elsewhere.^24^ The study received an approval by the local Institutional Review Board.

### Inclusion and exclusion criteria

The study's inclusion criteria were: 18–44 years of age, self-identified as Black/African American or white, being overweight or obese before pregnancy (BMI ≥25 kg/m^2^ and weight ≤370 lbs), ≤16 weeks gestation, and no intention of moving from the study area within the next 18 months. Women were ineligible if they had any of the following: uncontrolled hypertension or thyroid disease, insulin-dependent diabetes, hospitalization for a mental health or substance abuse disorder in the past six months, multiple gestation, persistent bleeding in the first trimester, history of more than three miscarriages, current or previous eating disorder, history of incompetent cervix, any physical disability that prevents exercise, doctor's advice not to exercise during pregnancy, irregular or inconsistent phone access, unwillingness to take part in intervention components (telephone calls) or randomization, and any medical condition not outlined above that the study medical monitor believed created an unsafe condition for participation. Women with a nickel allergy were originally excluded because the physical activity monitor contained this metal, but they were later included due to the availability of nickel-free activity monitors.

Finally, women who were not available to attend in-person group sessions were excluded initially. Due to the challenges in recruiting multiple women at one time to form a group and the less-than-ideal attendance at sessions, a protocol change was made in June 2015 (the sixth month into recruitment) to replace these 10 group sessions with 10 individual phone counseling calls, allowing for rolling recruitment. Only one intervention group was conducted (*n* = 6).

### Recruitment process

We used a two-step screening process. First, women underwent a seven-item initial screening at the obstetric clinic or through a website questionnaire, which assessed inclusion criteria based on age, race, gestational age, prepregnancy body mass index (BMI), and plans to move out of the study area. The initial screening form also asked for contact information and permission to contact for further screening. Second, study staff called those who were potentially eligible based on initial screening form. If the woman could not be reached through phone, contact was made through email or text message. A maximum of five attempts were made to reach women for secondary screening.

Women who remained eligible after the secondary screening were then guided through an interview in which they explored the expectations of participation in each study arm and potential benefits of participation in each study arm. Participants were asked to identify any perceived barriers to participation in each study arm, and potential ways to overcome these barriers. This interview was modeled after a protocol described by Goldberg and Kiernan,^25^ based on principles of motivational interviewing. The purpose of the interview was to ensure that participants made an informed decision to enter the study and to enhance later retention. Those who remained interested and eligible for the study answered additional questions related to our intervention approach, including information about their access to landline, cell phone, smart phone, computers, and their experience of using Facebook and podcasts. Lastly, participants were scheduled for a baseline measurement visit.

### Baseline visit and randomization

At the baseline visit, participants completed an informed consent form followed by interviewer-administered questionnaires that collected sociodemographic, behavioral, psychosocial, and health (before and during pregnancy) data, as well as interviewer-assessed measures of weight, height, and blood pressure. All women completed the first 24-hour dietary recall at this visit and the second dietary recall on a randomly selected day within 7 days of the visit either online or through phone with study staff. If they did not complete the second diet recall when scheduled, staff randomly assigned another date within 10 days from their originally scheduled second recall to complete it. Moreover, women were instructed about how to wear a SenseWear armband to collect physical activity and sleep data and how to return it through mail. Women who wore the armband for ≥5 days for ≥21 hours per day were considered compliant. They were given the opportunity to rewear the monitor if they did not meet criteria for compliance.

Only those women who completed all baseline measurement activities and were compliant with the two 24-hour dietary recalls and armband data collection were randomized and enrolled into the study. The study coordinator randomized participants using a randomization list generated by the statistician.

### Data analyses

We summarized our recruitment strategies, recruitment yields by recruitment approaches, and main reasons that women were excluded from the study. We also examined characteristics of women who were randomized versus those who were not to better understand the factors affecting participation among those eligible for inclusion. Characteristics studied were gestational age at phone screening, prepregnancy BMI and weight status, and chronic hypertension. In addition, we compared these two groups by additional variables relevant to the intervention approach (*e.g.*, access to phone, computers, and Facebook and podcast uses). Chi-square tests of independence or the two-sided Fisher's exact tests were used for categorical variables, and two-sample *t*-tests were used for continuous variables. All analyses were conducted in SAS version 9.4 (SAS Institute, Inc., Cary, NC).

## Results

### Recruitment strategies

Table 1 summarizes our recruitment strategies. Recruitment was done through direct and indirect recruitment from the obstetric clinics, and self-referred recruitment. Direct recruitment was defined as study staff recruiting in-person at the obstetric clinics. HIPP recruitment staff approached new obstetric patients either in the waiting room or before their first ultrasound to determine their interest in participating in the study. Interested patients filled out the screening form to determine initial study eligibility.

**Table 1.** Strategies to recruit pregnant women with elevated body weight (body mass index ≥25 kg/m^2^)

• Research staff conduct screening activities at obstetric clinics
• Clinical staff screen patients at the clinics
• Distribute flyers at obstetric and pediatric clinics, prenatal care, childcare center, local governmental offices (*e.g.*, WIC, family planning, immunization programs)
• Distribute flyers through mailing list or newsletters
• Use paid Facebook advertisement for targeted audience
• Local events or fairs such as baby cloth consignment, etc.
• Local magazines or newsletters
• Solutions to eliminate barriers for participants, for example, conducting home visits, offering after-hour or weekend clinic visits, providing childcare at study visits, offering bus tickets, etc.

WIC, Women, Infants, and Children Nutrition Program.

Indirect recruitment was defined as recruitment conducted by clinic or program staff. In 11 clinics and 1 Healthy Start program site, clinic staff screened patients for the study. Research staff first met with nursing managers at these clinics to identify the most efficient and feasible protocols that fit the busy clinic flow. Some clinics preferred to have the front desk staff complete the screening, whereas other clinics preferred to have medical assistants (before their ultrasonic exam to confirm the pregnancy) or nurses complete the screening. Study staff trained the clinic staff to give all new obstetric patients a brochure, briefly review the study with the patient, and ask the patient to complete a screening form if interested. Patients could also review the brochure later and call study staff if interested. Study staff collected screening forms from clinics weekly.

To show appreciation to clinic staff, we provided breakfasts, holiday gift baskets, or ice cream parties once or twice a year regardless of recruitment yield. In the last two years of recruitment, we also provided small incentives (gift cards) based on the number of screening forms returned to the study, irrespective of eligibility or randomization outcomes.

Furthermore, we recruited self-referred participants by advertisements. We posted and distributed flyers in locations where pregnant women typically frequent, including obstetric and pediatric clinics, prenatal classes, local governmental health service sites (such as WIC [the Women, Infants, and Children Nutrition Progrma], family planning, and immunization programs), childcare centers, and the University where the study took place. Study information was also included in mailing lists or newsletters of large employers. Furthermore, we advertised on Facebook using paid targeted advertisements. Advertisements were run bimonthly and targeted accounts of female users based on geographic “current city” location (proximity to the study area), age, and interest in pregnancy and parenting topics on Facebook. The targeted advertisement approach was efficient considering low staff efforts.

A link to an online screening form was included in flyers, emails, newsletters, and Facebook advertisements. Responses were reviewed by study staff on an incoming basis, and attempts were made to contact women who were initially eligible.

### Recruitment yields

**FIG. 1.**
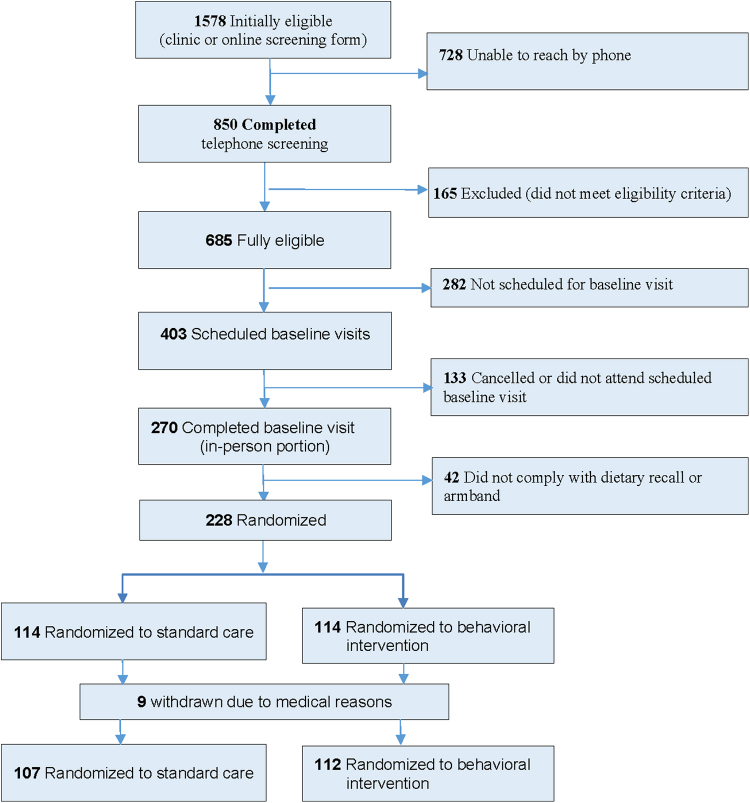
Study recruitment.

As shown in Figure 1 and Table 2, 1578 women were initially eligible. Of these women, 41.8% (659) were identified through direct recruitment by study staff in obstetric clinics, 51.9% (820) were identified indirectly by clinic staff, and 6.3% (99) were self-referred. Of those self-referred, 60.6% (60) responded to paid Facebook advertisements, whereas 39.4% (39) responded to flyers (17), were referred by someone they knew (8), heard about the study from an online source (4), reported some other source (4), or did not report how they heard about the study (6).

**Table 2.** Recruitment yields from direct, indirect, and self-referred (including Facebook) approaches by major milestones of recruitment

Recruitment approach	Initially eligible, n (%)	Completed telephone screening, n (%)	Fully eligible, n (%)	Scheduled baseline visits, n (%)	Completed baseline visits (in-person portion), n (%)	Randomized, n (%)
Total, *N* (%)	1578 (100.0)	850 (100.0)	685 (100.0)	403 (100.0)	270 (100.0)	228 (100.0)
Direct	659 (41.8)	364 (42.8)	308 (44.9)	181 (44.9)	132 (48.9)	110 (48.2)
Indirect	820 (51.9)	419 (49.3)	322 (47.0)	183 (45.4)	108 (40.0)	94 (41.2)
Self-referred total^a^	99 (6.3)	67 (7.9)	55 (8.0)	39 (9.7)	30 (11.1)	24 (10.5)
Facebook paid ads	60 (60.6)	39 (58.2)	32 (58.2)	20 (51.3)	15 (50.0)	6 (40.0)
Other sources	39 (39.4)	28 (41.8)	23 (41.8)	19 (48.7)	15 (50.0)	9 (60.0)

^a^ Self-referred participants were categorized by the sources where they heard about the study. These included Facebook paid advertisements and others such as flyers, online search, referred by someone they know, others, or no answers.

Nearly 54% (850) of initially eligible women were reached by telephone to complete the second eligibility screening. Of those reached, 42.8% (364) were from direct recruitment, 49.3% (419) from indirect recruitment, and 7.9% (67) from self-referred recruitment (58.2% from Facebook advertisement). A total of 80.6% (685) of those reached were eligible to participate in the study. After the motivational interview, 58.8% (403) remained interested and were scheduled for a baseline visit, whereas 41.2% (282) were not interested in participating the study or were not ready to schedule a baseline visit. Two-thirds (66.9%, 270) of those scheduled for a baseline visit completed the visit, whereas one-third (33.1%, 133) cancelled or did not attend their scheduled baseline visit. Among those who completed a baseline visit, 15.6% (42) did not comply with the diet or armband assessment.

As a result, 228 women were randomized, representing 33.3% (228/685) who were eligible for the baseline visit after telephone screening and 14.4% (228/1578) of those initially eligible based on the brief, seven-item screening measure. Of those randomized, 48.2% (110) were from direct recruitment, 41.2% (94) from indirect recruitment, and 10.5% (24) from self-referred recruitment (40% being from Facebook Advertisement).

### Main reasons for being excluded

Table 3 presents the reasons why women were excluded from the study during the telephone screening. The most common reasons, reported by at least 20 women, were a history of incompetent cervix, insulin-dependent diabetes, or a doctor's recommendation of no exercise during pregnancy.

**Table 3.** Reasons for exclusions reported during the telephone interview (*N* = 165)

Reasons	n (%)^a^
History of incompetent cervix	26 (15.7)
Insulin-dependent diabetes	23 (13.9)
Doctor's advice to not exercise during pregnancy	20 (12.1)
Moving out of the study area in the next 18 months	17 (10.3)
Gestational age >16 weeks at screening	15 (9.0)
History of more than three miscarriages	15 (9.0)
Persistent bleeding in first trimester	13 (7.9)
Nickel allergy^b^	12 (7.2)
Eating disorder or malnutrition	10 (6.0)
Not available to attend group sessions^b^	9 (5.4)
Physical disabilities that prevent exercising	7 (4.2)
Not willing to complete 10–20-minute phone calls weekly or biweekly	7 (4.2)
Blood pressure not controlled	6 (3.6)
Pregnant with more than one baby	6 (3.6)
Uncontrolled/untreated thyroid disease	5 (3.0)

^a^ Some participants were not eligible for multiple reasons. Only those conditions with ≥5 women reporting are presented. Thus, the numbers and percentages do not sum to 100%.

^b^ These two reasons were initially criteria for ineligibility, but these criteria were removed later in the study.

### Characteristics of eligible women

Eligible women had a mean gestational age of 10.2 weeks (SD = 2.6) at screening. Their mean prepregnancy BMI was 32.5 kg/m^2^ (SD = 6.2), 56.1% were obese, 43.9% were overweight, and 12.2% had hypertension (Table 4). Questions regarding phone access and social media use were only asked of those who were still interested in participating after discussing specifics of the study (*n* = 403). Over 95% of these women had a cell phone, a smart phone, or access to a computer with internet at home or at work. The majority of those with a smartphone had a monthly/contract plan (77.3%) and an unlimited data plan (67.5%). Regarding Facebook use, 91.6% had a Facebook account and logged onto their Facebook on average 5.9 days (SD = 1.9) in the past week with an average of 5.2 times per day (SD = 8.5). They reported posting on their Facebook account or responding to others an average of 7.3 times per week (SD = 14.6). Half (49.9%) of the women reported to have ever downloaded a podcast. Only 16.6% reported ever downloading a health-related podcast before the study.

**Table 4.** Characteristics of women who were eligible, overall, and differences by randomization status

	Eligible women	Randomized	Not randomized	p^a^
Total initially eligible women, *N* (%)	685 (100.0)	228 (33.2)	457 (66.7)	
Gestational age at screening in weeks, mean (SD)	10.2 (2.6)	10.1 (2.5)	10.6 (3.0)	**0.03**
Prepregnancy BMI, mean (SD)	32.5 (6.2)	32.3 (5.9)	32.6 (6.1)	0.58
Prepregnancy overweight status, %				
Overweight (BMI: 25.0–29.9)	43.9	48.2	41.9	0.12
Obese (BMI: ≥30.0)	56.1	51.8	58.1	
Self-reported hypertension, %	12.2	14.9	10.9	0.13
Eligible and interested women,^b^*N* (%)	402 (100.0)	227 (56.5)	175 (43.5)	
Have a home phone (landline), %	15.4	14.9	16.0	0.78
Have a work phone, %	25.1	25.3	24.7	0.89
Have a cell phone, %	99.0	100.0	97.7	**0.04**^a^
Receive ≥20 text messages daily, %	41.5	37.2	46.8	**0.05**
Share cell phone with a friend/family member, %	5.6	3.1	8.6	**0.02**
Have a smart phone, %	97.6	98.3	96.8	0.36^a^
Have a monthly/contract plan, %	77.3	80.2	73.8	0.12
Have unlimited data plan, %	67.5	62.8	73.2	**0.03**
Have access to a computer with internet, %	95.9	97.8	93.7	**0.04**^a^
Home, %	94.1	96.4	91.3	**0.03**^a^
Work, %	61.3	67.8	53.3	**0.003**
Have a Facebook account, %	91.6	92.5	90.5	0.46
No. of days log onto Facebook last week, mean (SD)	5.9 (1.9)	6.1 (1.8)	5.8 (2.0)	0.24
No. of times/day log onto Facebook, mean (SD)	5.2 (8.5)	4.4 (5.6)	6.2 (10.9)	**0.04**
No. of times post/week on Facebook, mean (SD)	7.3 (14.6)	6.7 (14.7)	8.2 (14.5)	0.31
Ever downloaded a podcast, %	49.9	55.5	43.2	**0.01**
Ever downloaded a health-related podcast, %	16.6	15.0	18.5	0.34

^a^ *p*-Values from two-sided Fisher's exact tests when the cell size was <5. Otherwise, *p*-values were from chi-square tests of independence for categorical variables and two-sampled *t*-tests for continuous variables. *p*-Values in bold face were statistically significant at the 0.05 level.

^b^ Sample size was smaller because the information below was asked for those who were still interested in participating after going through an interview about the specifics of the study. Some variables may be smaller than this due to missing data.

BMI, body mass index; SD, standard deviation.

### Comparison of eligible women who were randomized versus not randomized

Women who were randomized were slightly earlier in their pregnancy in terms of gestational age at screening, compared with women who were not randomized. They did not differ by prepregnancy BMI, weight status, or having hypertension. Randomized participants were more likely to own a cell phone (100.0% vs. 97.7%), have access to a computer with internet (97.8% vs. 93.7%), have internet access at home (96.4% vs. 91.3%) and work (67.8% vs. 53.3%), or have ever downloaded a podcast (55.5% vs. 43.2%). However, they were less likely to report receiving ≥20 text messages daily (37.2% vs. 46.8%) and sharing a cell phone with a friend or family member (3.1% vs. 8.6%), and they logged onto Facebook less frequently (4.4 vs. 6.2 times/day). Women who were randomized were less likely to have unlimited data plan if they owned a smartphone (62.8% vs. 73.2%) (Table 4).

### Characteristics of women randomized into the study

Among randomized participants, 55.7% were white and 44.3% were African American. They were nearly equally split among overweight (48.4%) and obese (51.6%) weight categories. Participants averaged 29.7 years of age (range: 18–42), two-thirds were married, and 42.9% were nulliparous. Nearly 60% of participants were college educated, 37.6% had a family annual income greater than $75K, 61.2% were full-time employed, and 30.0% were covered by Medicaid. Randomized participants averaged 12.5 weeks of gestation at the baseline visit (range: 6.9–18.7 weeks) (Table 5).

**Table 5.** Characteristics of enrolled study participants (*N* = 219)

	N (%)
Prepregnancy BMI	
Overweight	106 (48.4)
Obese	113 (51.6)
Race	
White	122 (55.7)
African American	97 (44.3)
Age	
18–24	35 (15.9)
25–29	62 (28.3)
30–34	82 (37.4)
≥35	40 (18.3)
Marital status	
Married	147 (67.1)
Unmarried^a^	72 (32.9)
Parity	
0	94 (42.9)
1	80 (36.5)
≥2	45 (20.6)
Education	
Grades 9–11	3 (1.4)
Grade 12 or GED	24 (10.9)
College 1–3 years	62 (28.3)
College 4 years or more	135 (59.5)
Family income	
< $35,000	64 (29.4)
$35,000–$49,999	30 (13.8)
$50,000–$74,999	42 (19.3)
≥ $75,000	82 (37.6)
Employment during pregnancy	
Employed full-time	134 (61.2)
Part-time or unemployed	85 (38.8)
Medicaid recipients	
Yes	66 (30.1)
No	153 (69.9)
	**Mean (SD)**
Prepregnancy BMI	32.3 (5.9)
Age, years	29.7 (5.0)
Gestational age at baseline, in weeks	12.6 (2.3)

^a^ Unmarried included divorced (*n* = 8), separated (*n* = 7), and never married (*n* = 57) women.

## Discussion

Our recruitment yield was low, and we did not meet the study recruitment target. Only 14% of those initially eligible or 33% of those eligible after further screening were randomized, but this yield was slightly higher than the recruitment yield (25% of those who were eligible) in the LIFE-Moms consortium trials.^19^ Recruiting overweight or obese pregnant women in early pregnancy into a behavioral lifestyle intervention proved challenging.

Our low recruitment rate is not surprising given various challenges that pregnant women face in this special life stage. Early pregnancy is a time when women may experience pregnancy-related discomfort, fatigue, and nausea that may make them reluctant to take on more responsibilities (*e.g.*, the commitment of participating in an 18-month study). This reluctance could explain why 40% of eligible women were not interested in participating in our study after motivational interviews. Although motivational interviewing may have adversely impacted recruitment, we believe that this interview may have contributed to our favorable retention rates after enrollment (87% at 32 weeks gestation, 79% at 6 months postpartum, and 77% at 12 months postpartum). Pregnancy presents a unique challenge for recruitment because it is a temporary and time-sensitive state, which may disqualify initially eligible women as they progress in their pregnancy (*e.g.*, exceeding enrollment age <16 weeks of gestation, pregnancy losses, multiple pregnancies, or developing complications that are contraindicating with exercise during pregnancy).

Second, this low recruitment yield also reflects the usual challenges in randomized controlled trials. For example, some women were motivated to participate in our study because of their interest in joining only either the intervention group or standard care group. The randomized design and inability to know which group they would be assigned to might have led to some women declining participation. Finally, we faced various logistic changes in our collaborating clinics such as staff turnover, leadership change, clinic relocation, and holiday or weather-related closures. These unplanned events paused our recruitment efforts for periods of time.

Several studies have reported recruitment yields and strategies or lessons for recruiting pregnant women for behavioral lifestyle interventions.^20,26–29^ Consistent with other studies,^20,26,29^ we also found that strategies such as in-person meetings and close relationships with providers, clinics and participants enhanced recruitment. Our study also supports recommendations made by Maghera et al.^27^ that diverse recruitment methods should be used and social media, especially paid-media advertisement, is a promising method for recruitment. In our study, 10% of our final sample was recruited through self-referral and 40% of those self-referred were from Facebook. Compared with other advertisement channels, Facebook had the highest yield.

Furthermore, a Cochrane review^16^ recommended that future studies pay attention to innovative interventions utilizing mobile phone technology. Our study delivered the intervention through both traditional intervention channels (*i.e.*, in-person and telephone-based) and more innovative channels (*i.e.*, podcasts and Facebook support). We found that although access to cell phones, computers with internet at home or at work, and Facebook was high, women who were not randomized into the study had slightly less access to these technologies. Our study population had a moderate level of experience with podcasts, yet a higher percentage of randomized women reported having ever downloaded a podcast than standard care women. Our results indicate that these technologies can be integrated into future interventions, although technologies (especially podcasts) might reduce recruitment yield.

We learned several facilitators and strategies in recruiting pregnant women. First, it is essential to have a network of obstetric clinical partners who are supportive of research studies in pregnancy and are willing to collaborate with the research team to identify and reduce clinic-related barriers to patient recruitment. In our study, nearly 94% of screened pregnant participants were derived from obstetric clinics. Depending on the obstetric clinic's preferences, the front desk staff, medical assistants, laboratory technicians, and/or nursing staff helped us identify potentially eligible participants.

Second, building relationships and conducting on-site recruitment were time consuming, requiring patience, and effective communication skills from recruitment staff. Yet based on our experience, the direct recruitment by research staff was more reliable than depending on clinic's staff to conduct screening for the study. Many clinics were not able to consistently conduct screening for the study.

Third, other indirect approaches (*e.g.*, posting flyers, Facebook ads) are low-cost and worth considering in combination with clinic-based approaches for recruitment. Paid Facebook advertisements with targeted audiences in a geographic area resulted in a larger number of randomized participants than flyers. We found, however, that Facebook advertisements often did not result in further recruitments after one to two months of advertisement. This drop off may be due to specific parameters set on the advertisement algorithm not leading to new views. For example, if a Facebook user became pregnant, but did not search for parenting or pregnancy-related content, the advertisement might not have reached that user. Thus, we alternated the months with advertisement and months without to save funds.

Fourth, other strategies were used to eliminate barriers for participation. For example, we offered the option of conducting home visits (10.2% of pregnancy visits and 42.6% of postpartum visits), offered after-hour (11.9% of all visits) or weekend (6.5% of all visits) clinic visits, provided childcare for women with younger children at study visits (numbers not tracked), and offered bus tickets as a public transportation option if participants were without personal transportation (numbers not tracked).

One limitation of this study is that we did not conduct qualitative interviews to identify reasons underlying women's refusal to participate in this study among those who were eligible. We speculate that some women may be uninterested in research studies or unwilling to be randomized. Their reluctance might also be related to the aspects of the study such as 18-month follow-up, the frequency of intervention activities, and/or number or duration of measurement sessions. This study recruited participants who were more educated or with a lower proportion of them being on Medicaid than the general population in SC, thus limiting generalizability.^30^

Due to our persistence in recruitment and use of creative strategies, we recruited and randomized 219 overweight or obese pregnant women into the study. According to Muktabhant et al.^16^ only 4 out of 24 randomized controlled trials of overweight and/or obese women reached or exceeded a sample size of 200 women. They were conducted in Australia (*n* = 1),^31^ Denmark (*n* = 2),^32,33^ and the USA (*n* = 1).^34^ Another recently completed U.S. study, LIFE-Moms, was able to enroll 1150 overweight and obese pregnant women through 7 different trials, although most of these 7 trials did not reach their enrollment target.^19^ Furthermore, a systematic review of antenatal lifestyle interventions pointed out that existing trials are limited due to their inclusion of predominantly white samples.^17^ Thus, by recruiting a racially diverse sample (56% white and 44% African American women), our study fills a critical gap in literature.

## Conclusions

Recruiting overweight and obese women in early pregnancy for a behavioral lifestyle intervention was challenging. Our strategies and lessons learned offer valuable information for future research studies' planning and implementation. We recommend that future studies wishing to reach an obstetric population recruit from large obstetric clinics that can allocate resources to consistently support research recruitment or allow the research staff to recruit on site directly. The recruitment efforts can be supplemented by paid or free advertisements. These studies should be prepared for a lengthy duration of recruitment. Additionally, studies should identify reasons for refusal or non- or low participation in low-income participants, which will offer insights about how to recruit a representative sample. Identification of recruitment barriers and enablers may assist the participation of pregnant women with elevated BMI into future behavioral lifestyle intervention programs and support progress toward improving maternal/infant wellbeing.

## Abbreviations Used

BMI: body mass index
GWG: gestational weight gain
HIPP: Health in Pregnancy and Postpartum
IOM: Institute of Medicine
SD: standard deviation
WIC: Women, Infants, and Children Nutrition Program
